# Impact of COVID-19 on Children's and Adolescent's Mental Health in Saudi Arabia

**DOI:** 10.7759/cureus.19786

**Published:** 2021-11-21

**Authors:** Rheem A Almhizai, Sara H Almogren, Norah A Altwijery, Basim A Alanazi, Nora M Al Dera, Sarah S Alzahrani, Sara M Alabdulkarim

**Affiliations:** 1 Pediatric Medicine, Imam Mohammad Ibn Saud Islamic University, Riyadh, SAU; 2 Medicine, Imam Mohammad Ibn Saud Islamic University, Riyadh, SAU

**Keywords:** mental health, adolescents, children, saudi arabia, covid-19

## Abstract

Background

COVID-19 outbreak was sudden and unexpected in most countries. It has spread globally between January and March 2020. The World Health Organization (WHO) first declared the outbreak as a global pandemic on 11 March 2020. While this lockdown has proven to be an important and successful method of social distancing to counter the growing spread of the highly contagious COVID-19 virus, it has also created a degree of psychological impact on the public. Children may be strongly exposed to pandemic-generated biopsychosocial stressors, and once the containment measures of the population are needed to minimize the spread of viruses, they may be negatively impacted by the disturbance of everyday life as a result of social isolation. During school closures, children's routines may change, and healthy behaviors, such as physical activity, adequate diet, or good sleeping habits, may be less likely to happen.

Method

A cross-sectional study was conducted in Saudi Arabia from 20 March 2021 to 30 March 2021, targeted at children's parents and adolescents. Our concentration was on the impact of COVID-19 on the psychiatric wellbeing of children and adolescents. The data was collected through an online self-administered questionnaire which contains 56 close-ended questions for parents of children and 46 close-ended questions for adolescents. Statistical analysis was performed using R v 3.6.3 (R Foundation, Vienna, Austria). Counts and percentages were used to summarize the distribution of categorical variables.

Results

The questionnaire was completed by 1141 respondents, 454 were < 18 years old. Thus, these respondents completed the questionnaire on behalf of themselves. The remaining 688 respondents were adults and completed the questionnaire on behalf of their children. Results showed that higher children's age was associated with less increase in worrying, restlessness, and a higher increase in sadness. Higher age was associated with a higher increase in the frequency of waking up, sleeping little, and uneasiness, and nervousness. Having relatives who were infected with COVID-19 was associated with higher increases in most of the negative behaviors such as anxiety, sadness, sleeping little, indecisiveness, and irritability. Punishment threats, screaming, and hitting were associated with a higher increase in negative behaviors during the pandemic compared to before the pandemic. Results showed that neither the gender of the parent nor the child was associated with any of the domains of the child's behavior. Children whose parents were divorced had higher scores on anxiety, restlessness, and sleep disorders than children whose parents were not divorced

Conclusion

COVID-19 has caused increased stress on families, especially children and adolescents who are vulnerable populations. Our results show that the COVID-19 pandemic can affect the mental health of children and adolescents in Saudi Arabia. We showed that parental stress is a predictor of psychiatric problems, which, if unaddressed, can cause child maltreatment and greater psychological distress.

## Introduction

The outbreak of the 2019 coronavirus disease (COVID-19) began in Wuhan City, China, in December 2019 [1]. The outbreak was sudden and unexpected in most countries. It has spread globally between January and March 2020. The World Health Organization (WHO) first declared the outbreak as a global pandemic on March 11, 2020.

The current COVID-19 pandemic has become a challenge to psychological health as previous studies revealed a deep and wide spectrum of psychosocial effects during past outbreaks of infectious diseases on human, community, and international levels. [2] Since then, regional isolation measures or lockdowns have begun to be enforced in Saudi Arabia, among other countries. Children may be strongly exposed to pandemic-generated biopsychosocial stressors, and once the containment measures of the population are needed to minimize the spread of viruses, they may be negatively impacted by the disturbance of everyday life as a result of social isolation [3,4]. In this context, school closures, social distancing, and home quarantine were some of the key steps taken during the lockdown to prevent the transmission of this infection [5]. Children may be strongly exposed to pandemic-generated biopsychosocial stressors, and once the containment measures of the population are needed to minimize the spread of viruses, they may be negatively impacted by the disturbance of everyday life as a result of social isolation [3,4]. While this lockdown has proven to be an important and successful method of social distancing to counter the growing spread of the highly contagious COVID-19 virus, it has also created a degree of psychological impact on the public [6]. Everything changes among children; they cannot play outside as they did before. When a child walks outside, they must wear a face mask. It is difficult for youngsters to understand the rationale for wearing a face mask, or some children may understand the reason but still only wear it because their parents urge them to. It has been demonstrated that this pandemic may appear to have more long-term negative consequences on children and adolescents than on adults [7]. During school closures, childrens' routines may change, and healthy behaviors, such as physical activity, adequate diet, or good sleeping habits, may be less likely to happen [8]. The children now spend more time in the house. Daily habits change, the child who loved playing football, now sits in the house and plays it on the internet instead. The child may not have the coronavirus disease, but the impact of COVID-19 affects the child's health indirectly by affecting the life of the child. The severity of its impact on this age group depends on many factors, such as the age of development, the educational status, family income, pre-existing mental health conditions, and the child or family member being quarantined because of a COVID-19 infection [9]. COVID-19 outbreak can have many effects on the lives of children as well as young adolescents, such as chronic and acute stress, concern for their families, unexpected mourning, sudden school closures, and increased time on the internet and social media [10]. The lockdown affects the parents and puts stress on them because of the loss of jobs and social distance. That violates children at home. It has increased with the COVID-19 pandemic. There are 1.8 billion children in 104 countries who had no prevention service due to COVID-19 [11]. Children's emotional well-being has been impaired in many ways, as this COVID-19 situation has altered the way they usually grow, read, play, act, interact, and control emotions [8]. During this stressful time, children with pre-existing psychiatric disorders such as attention deficit hyperactivity disorder (ADHD), anxiety and depression could be adversely affected [12]. The mental development of a child has been found to be drastically and negatively affected if left untreated [1]. Mental health is a very well-established component of human development and determines the outcome of a child's educational achievements and the ability to live fulfilling and productive lives [13]. 

Study objective

The main objective of this study is to find out and understand the impact of COVID-19 on children's and adolescents' mental health. The study also aims to compare the mental health of children and adolescents before and after the pandemic, to analyze the factors contributing to behavioral and mental changes, and to raise awareness in parents and teachers about the impact of COVID-19 on children's and adolescents' mental health.

## Materials and methods

Study design

A cross-sectional study was conducted in Saudi Arabia from 20/3/2021 to 30/3/2021, targeted at parents of children and adolescents. Our concentration was on the impact of COVID-19 on the psychiatric wellbeing of children and adolescents. The data was collected through an online self-administered questionnaire which contains 56 close-ended questions for parents of children and 46 close-ended questions for adolescents) close-ended questions, which were guided by the study objective and review of the literature to assess the mental status of adolescents and children in the past few days of filling the survey (e.g., restless, anxious, and trouble sleeping, etc.). The questionnaire is an Arabic translated version distributed through the internet network (e.g., social media). The questionnaire included a set of questions about the socio-demographic characteristics of the respondents. Five questions were to assess the job security status. The second section of the questionnaire included four questions to assess the child's gender, age, and educational level. The third section included six questions about the family's behavior towards their children. The last part was a Likert scale conducted of 33 short questions evaluating the mental status of the child. All responses agreed to the informed consent, responses from parents of more than 18 years old children, and responses from non-Saudi parents and adolescents, and incomplete or duplicated forms were excluded from the study.

Ethical approval

This study has been approved by the university's IRB department at Imam Mohammad Ibn Saud Islamic University, College of Medicine.

Statistical analysis

Statistical analysis was performed using R v 3.6.3 (R Foundation, Vienna, Austria). Counts and percentages were used to summarize the distribution of categorical variables. The mean ± standard deviation was used to summarize the distribution of continuous variables. Chi-square test of independence was used to assess the association between categorical variables. Cronbach's alpha was used to assess the reliability of the 33 items related to children's behavior during the pandemic. Spearman's correlation was used to assess the association between parents' behavior and children's behavior during the pandemic. The association between relatives' exposure to Covid-19 and children's behavior was also assessed. Exploratory factor analysis (EFA) using Promax rotation was used to explore the underlying structure of the 33 items. Items with low loadings (< 0.3) or cross-loaded on more than one factor (difference in loadings < 0.3) were excluded from the EFA. Items belonging to each factor were averaged, and linear regression was used to assess the predictors of higher change. Independent factors included the socio-demographic characteristics of the parents and children. Hypothesis testing was performed at a 5% level of significance.

## Results

Descriptive statistics for the study sample

As illustrated in Table 1, the questionnaire was completed by 1141 respondents. Of these, 454 were < 18 years old (360 were aged 16 - 18 years old, and 94 were < 15 years old). Thus, these respondents completed the questionnaire on behalf of themselves. The remaining 688 respondents were adults and completed the questionnaire on behalf of their children.

**Table 1:** Descriptive statistics for the included caregivers (N=688)

Factors	N (%), N=688
Gender	
Female	512 (74.4%)
Male	176 (25.6%)
Age (years)	
From 19 to 25	104 (15.1%)
From 26 to 35	185 (26.9%)
From 36 to 45	240 (34.9%)
From 46 to 55	122 (17.7%)
More than 55	37 (5.38%)
Educational level	
Elementary and middle school	16 (2.33%)
High school	107 (15.6%)
Diploma	8 (1.16%)
Bachelor's degree	472 (68.6%)
Post-graduate education	85 (12.35%)
Marital status	
Divorced	65 (9.45%)
Married	600 (87.2%)
Widow	23 (3.34%)
Number of children	
One child	136 (19.8%)
Two children	131 (19.0%)
Three to five children	304 (44.2%)
More than five	117 (17.0%)
Economic status (Riyals)	
Less than 5000	132 (19.2%)
From 5000 to 10000	199 (28.9%)
From 10000 to 15000	178 (25.9%)
More than 15000	179 (26.0%)
Occupational status	
Employee	423 (61.5%)
Freelancer	52 (7.56%)
Non-employee	213 (31.0%)
Type of work	
Office place	216 (46.6%)
Working online from home	248 (53.4%)
Covid-19 had an impact on income	
No, it did not affect income	242 (35.2%)
Yes, it did	446 (64.8%)
Impact of Covid-19 on income	
Decreased income	327 (74.5%)
Increased income	112 (25.5%)
Probability of losing job during the pandemic	
High risk	161 (23.4%)
Low risk	143 (20.8%)
No risk of losing job during the pandemic	384 (55.8%)

In Table 1, 688 parents completed the questionnaire on behalf of their children (74.4% were mothers and 25.6% were fathers). The age of the included parents ranged from 19 - 25 years (15.1%) to > 55 years (5.38%). More than three-quarters of the parents completed university education (68.6% completed university education, and 12.35% completed a post-graduate degree. Approximately half of the respondents (44.2%) had three to five children, 19.8% had one child, and 19% had two children. All economic statuses were approximately equally represented in the study sample. Regarding occupational status, more than half of the respondents were employees (61.5%), 31% were non-employees, and 7.56% were freelancers. More than half of the respondents worked online from home (53.4%), and 46.6% worked from the office. More than half of the respondents thought that COVID-19 affected their income. Of these, 74.5% mentioned that the effect was negative, and 25.5% mentioned that the effect was positive. Half of the respondents thought that COVID-19 did not affect the probability of losing their job (55.8%), 20.8% thought that the probability was low, and 23.4% thought that the probability was high.

The majority of the respondents were from the central region (82.6%, Table 2). Male and female children represented 42.5% and 57.5% of the study sample. Approximately one-half (43.7%) of the respondents were aged 15 - 18 years old, and one-quarter (23.5%) were aged 6 - 11 years old. The remaining 10.3%, 12.7%, and 9.73% were aged 12 - 14, less than 3, and four for five years old, respectively. Regarding education, 40.1% of the children were attending high school, 24% were attending elementary school, and 13.9% attended middle school. A total of 17% and 4.91% of the children were preschoolers or attended kindergarten, respectively. Approximately three-quarters (69.1%) of the respondents knew relatives/neighbors who were infected with COVID-19. 76.2% of the parents thought that they took action to keep the child busy during the lockdown. More than half of the respondents thought that the child was acting normally as before the lockdown (59.7%). One-quarter of the respondents thought that the family was busy with work during the lockdown, 27.7% threatened their children with punishment for improper behavior during the lockdown, and 37.2% of the parents screamed at their children during the lockdown. A total of 190 (16.7%) parents hit their children during the lockdown.

**Table 2:** Descriptive statistics for the included children (N=1141)

Factors	N (%), N=1141
Region	
Central region	942 (82.6%)
Eastern region	70 (6.13%)
Northern region	36 (3.16%)
Southern region	41 (3.59%)
Western region	52 (4.56%)
Gender	
Female	656 (57.5%)
Male	485 (42.5%)
Age (years)	
3 years or less	145 (12.7%)
From 4 to 5	111 (9.73%)
From 6 to 11	268 (23.5%)
From 12 to 14	118 (10.3%)
From 15 to 18	499 (43.7%)
Educational level	
Elementary school	274 (24.0%)
High school	458 (40.1%)
Kindergarten	56 (4.91%)
Middle school	159 (13.9%)
Preschool	194 (17.0%)
Relatives/neighbors infected with Corona	
No	352 (30.9%)
Yes	789 (69.1%)
Family took any action to keep the child busy during lockdown	
No	272 (23.8%)
Yes	869 (76.2%)
Child acting normal as before lockdown	
No	460 (40.3%)
Yes	681 (59.7%)
Family is busy with work during lockdown	
No	842 (73.8%)
Yes	299 (26.2%)
Threatened child with punishment for improbity during lockdown	
No	825 (72.3%)
Yes	316 (27.7%)
Family member screamed at a child during lockdown	
No	716 (62.8%)
Yes	425 (37.2%)
Family member hit a child during the lockdown	
No	951 (83.3%)
Yes	190 (16.7%)

**Figure 1:**
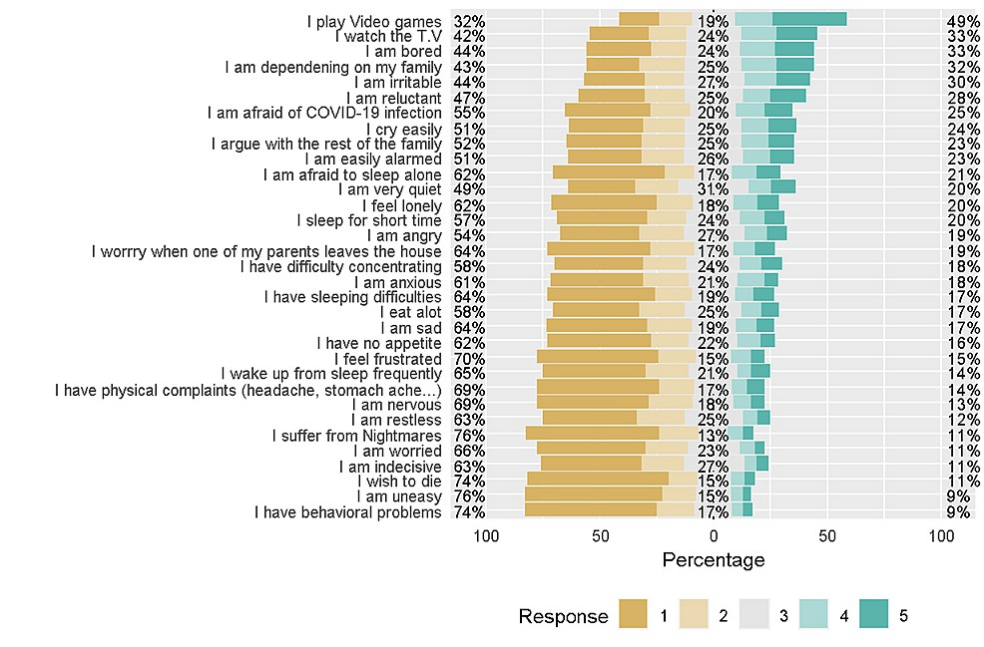
Perception of the emotional and behavioral effects of the quarantine in their children 1 = never, 2 = rarely, 3 = sometimes, 4 = often, 5 = always

Figure 1 shows that only 9% of the respondents thought that their or their children's behavioral problems during the quarantine were higher than before quarantine. A similar pattern was observed for uneasiness. Approximately half of the respondents thought that they or their children played video games to a greater extent during the quarantine than before the quarantine, and one-third watched TV to a greater extent during the quarantine than before the quarantine, and a similar percent were bored to a greater extent during the quarantine. The reliability of the 33 items was 0.95, which was greater than 0.7 and indicated good reliability of the items.

Results in Table 3 showed that higher children's age was associated with less increase in worrying (r = -0.119, P < 0.001), restlessness (r = -0.118, P < 0.001), and higher increase in sadness (r = 0.125, P < 0.001). Higher age was associated with higher increase in the frequency of waking up (r = 0.076, P < 0.05), sleeping little (r = 0.058, P < 0.05), and uneasiness (r = 0.082, P < 0.01), and nervousness (r = 0.122, P < 0.001). Having relatives who were infected with COVID-19 was associated with higher increase in most of the negative behaviors such as anxiety (r = 0.085, P < 0.01), sadness (r = 0.085, P < 0.01), sleeping little (r = 0.074, P < 0.01), indecisiveness (r = 0.073, P < 0.05), and irritability (r = 0.12, P < 0.001). Punishment threats, screaming, and hitting were associated with higher increase in the negative behaviors during the pandemic compared to before the pandemic. The correlation coefficients for screaming and hitting children (by their parents) were higher (for most of the behaviors) than other factors such as age, complaints of busy parents, and having relatives who were infected with COVID-19.

**Table 3:** Correlation between parents' behavior and the change in children's behavior during the pandemic

	Age	Relatives infected	Complaints of busy parents	Threat of punishment	Screaming	Hitting a child
My child is worried	-0.119^***^	0.047	0.068^*^	0.142^***^	0.159^***^	0.131^***^
My child is restless	-0.118^***^	0.008	0.010	0.249^***^	0.264^***^	0.234^***^
My child is anxious	0.030	0.077^**^	-0.035	0.185^***^	0.204^***^	0.193^***^
My child is sad	0.125^***^	0.085^**^	-0.074^*^	0.152^***^	0.201^***^	0.196^***^
My child has nightmares	0.026	0.085^**^	-0.104^***^	0.236^***^	0.233^***^	0.313^***^
My child is reluctant	-0.030	0.113^***^	-0.002	0.146^***^	0.298^***^	0.172^***^
My child feels lonely	0.031	0.106^***^	-0.070^*^	0.199^***^	0.248^***^	0.224^***^
My child wakes up frequently	0.076^*^	0.111^***^	-0.021	0.127^***^	0.153^***^	0.198^***^
My child sleeps little	0.058^*^	0.074^*^	-0.024	0.128^***^	0.185^***^	0.179^***^
My child is very indecisive	0.052	0.073^*^	-0.061^*^	0.180^***^	0.184^***^	0.206^***^
My child is uneasy	0.082^**^	0.058	-0.125^***^	0.242^***^	0.247^***^	0.304^***^
My child is nervous	0.122^***^	0.088^**^	-0.077^**^	0.227^***^	0.253^***^	0.297^***^
My child is afraid to sleep alone	-0.275^***^	0.086^**^	0.094^**^	0.214^***^	0.207^***^	0.253^***^
My child argues with the rest of the family	-0.018	0.023	0.041	0.188^***^	0.297^***^	0.165^***^
My child is very quiet	0.097^***^	0.013	-0.026	-0.036	-0.036	0.001
My child cries easily	-0.026	0.135^***^	0.038	0.102^***^	0.155^***^	0.143^***^
My child is angry	-0.031	0.071^*^	-0.002	0.237^***^	0.298^***^	0.244^***^
My child asks about death	0.104^***^	-0.000	-0.072^*^	0.218^***^	0.197^***^	0.265^***^
My child feels frustrated	0.188^***^	0.054	-0.123^***^	0.183^***^	0.212^***^	0.254^***^
My child is bored	0.021	0.126^***^	-0.001	0.146^***^	0.209^***^	0.145^***^
My child is irritable	0.014	0.120^***^	-0.017	0.138^***^	0.257^***^	0.175^***^
My child has sleeping difficulties	0.055	0.123^***^	-0.045	0.215^***^	0.224^***^	0.237^***^
My child has no appetite	-0.002	0.084^**^	0.037	0.192^***^	0.182^***^	0.189^***^
My child is easily alarmed	0.064^*^	0.120^***^	-0.011	0.140^***^	0.218^***^	0.171^***^
My child has difficulty concentrating	0.039	0.090^**^	-0.022	0.195^***^	0.247^***^	0.211^***^
My child is afraid of COVID-19 infection	0.021	0.043	0.124^***^	0.052	0.037	0.068^*^
My child is very dependent on us	-0.222^***^	0.108^***^	0.148^***^	0.107^***^	0.153^***^	0.117^***^
My child has physical complaints (headache, stomach ache ...)	0.194^***^	0.103^***^	-0.139^***^	0.140^***^	0.159^***^	0.165^***^
My child has behavioral problems	-0.001	0.028	-0.027	0.280^***^	0.215^***^	0.254^***^
My child eats a lot	0.157^***^	0.056	-0.052	0.046	0.107^***^	0.081^**^
My child worries when one of us leaves the house	-0.212^***^	0.029	0.092^**^	0.196^***^	0.123^***^	0.171^***^
My child watch T. V	-0.285^***^	-0.007	0.157^***^	0.148^***^	0.146^***^	0.143^***^
My child plays Video games	-0.050	0.052	0.118^***^	-0.028	0.085^**^	0.015
Statistical analysis was performed using Spearman's correlation* P < 0.05, ** P < 0.01, *** P < 0.0018						

**Figure 2:**
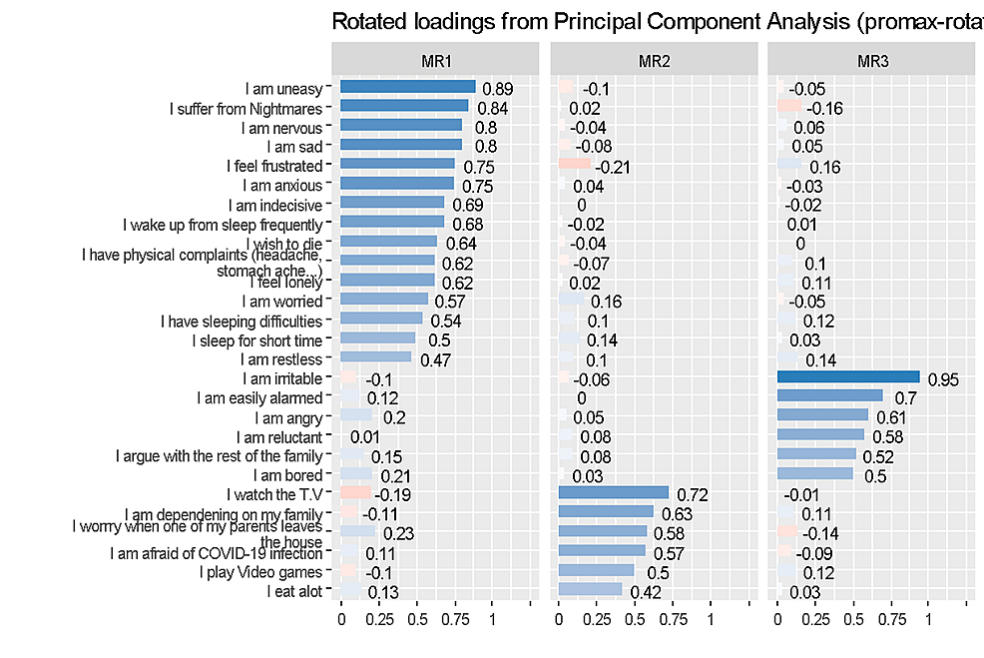
Exploratory factor analysis results

Results showed that three factors explained 27 items. Factor 1 included items related to anxiety, restlessness, and sleep disorders (n = 15). Factor 2 included items related to family dependency (n = 6). Factor 3 included items related to angriness and irritability (n = 6) as illustrated in Figure 2.

In Table 4, results showed that neither the gender of the parent nor the child was associated with any of the domains of the child's behavior. The age of the parent was not associated with any of the three factors (P > 0.05 for all three factors) except for family dependency, which was higher when parents were 46 - 55 years compared to when they were 19 - 25 years (B = 0.29, P < 0.05). Parents' education was not associated with any of the three domains of the child's behavior. Children whose parents were divorced had higher scores on anxiety, restlessness, and sleep disorders than children whose parents were not divorced (B = 0.23, P < 0.05). Children whose parents had more than one child reported significantly higher scores on the three domains for their children except when they reported having more than five children (P < 0.5). Better socioeconomic status (SES) was associated with higher scores on the anxiety, restlessness, and sleep disorders domain, with an average increase of 0.07 for each one-level increase in SES. Higher scores on all three domains were reported for parents who worked as freelancers compared to regular employees. Non-employment was associated with higher scores on the angriness and irritability domain (B = 0.37, P < 0.05) but not the remaining two domains. Children whose parents reported being affected by COVID-19 had higher scores on the three domains (P < 0.01 for all three domains). Similarly, children who knew relatives/neighbors infected with COVID-19 had higher scores on all three domains. Higher age was associated with a higher score on domains two (family dependency) and three (angriness and irritability), with an increase in the coefficient with the increase in age. This indicates that older children are more likely to be affected than younger children. Regarding school, children in elementary school were more likely to show family dependency during the pandemic than students in high school (B = 0.6, P < 0.05). Respondents in kindergarten and preschoolers were also more likely to show behavioral changes during the pandemic than students in high school.

**Table 4:** Factors associated with child's behavior during the pandemic Linear regression was used for the analysis Coefficients (B) represent the increase in score for each level change in the independent variable compared to the reference

	Anxiety, restlessness, and sleep disorders		Family dependency		Anger and irritability	
Predictors	Β (95% CI)	P-value	Β (95% CI)	P-value	Β (95% CI)	P-value
Parent's gender						
Female	Ref		Ref			Ref
Male	-0.06 (-0.22 – 0.09)	0.424	0.03 (-0.14 – 0.20)	0.696	-0.20 (-0.39 – 0.00)	0.053
Age	Ref		Ref		Ref	
From 26 to 35	-0.04 (-0.25 – 0.17)	0.711	0.09 (-0.14 – 0.32)	0.426	-0.13 (-0.40 – 0.14)	0.351
From 36 to 45	0.09 (-0.13 – 0.31)	0.435	0.18 (-0.07 – 0.42)	0.155	-0.10 (-0.39 – 0.18)	0.477
From 46 to 55	0.14 (-0.12 – 0.40)	0.279	0.29 (0.01 – 0.58)	0.041	0.08 (-0.25 – 0.41)	0.644
More than 55	0.01 (-0.34 – 0.37)	0.942	0.21 (-0.18 – 0.60)	0.283	-0.03 (-0.49 – 0.42)	0.883
Educational level						
University or higher	Ref		Ref		Ref	
High school/Less	0.08 (-0.08 – 0.25)	0.324	-0.17 (-0.35 – 0.02)	0.074	-0.08 (-0.29 – 0.14)	0.491
Marital status						
Married	Ref		Ref		Ref	
Widow	-0.19 (-0.53 – 0.16)	0.293	-0.13 (-0.51 – 0.25)	0.503	-0.05 (-0.49 – 0.40)	0.834
Divorced	0.23 (0.01 – 0.45)	0.041	0.10 (-0.14 – 0.34)	0.419	0.14 (-0.14 – 0.42)	0.319
Number of children						
One child	Ref		Ref		Ref	
Two children	0.14 (-0.09 – 0.36)	0.226	0.34 (0.09 – 0.58)	0.007	0.46 (0.18 – 0.75)	0.001
Three to five	0.23 (-0.00 – 0.46)	0.05	0.31 (0.06 – 0.56)	0.015	0.36 (0.07 – 0.66)	0.016
More than five	0.21 (-0.05 – 0.48)	0.117	0.17 (-0.12 – 0.46)	0.261	0.26 (-0.08 – 0.60)	0.131
Economic status	0.07 (0.01 – 0.14)	0.028	-0.02 (-0.09 – 0.05)	0.548	0.00 (-0.08 – 0.09)	0.917
Occupational status						
Employed	Ref		Ref		Ref	
Freelancer	0.22 (-0.02 – 0.47)	0.073	0.29 (0.02 – 0.55)	0.035	0.37 (0.06 – 0.69)	0.019
Unemployed	0.14 (-0.01 – 0.29)	0.064	0.03 (-0.13 – 0.19)	0.731	0.27 (0.08 – 0.46)	0.005
COVID-19 impact on income						
No	Ref		Ref		Ref	
Yes	0.29 (0.16 – 0.42)	<0.001	0.29 (0.15 – 0.44)	<0.001	0.23 (0.06 – 0.40)	0.007
Relatives/neighbors infected with COVID-19						
No	Ref		Ref		Ref	
Yes	0.18 (0.05 – 0.31)	0.007	0.29 (0.15 – 0.43)	<0.001	0.26 (0.09 – 0.43)	0.002
Child's gender						
Female	Ref		Ref		Ref	
Male	-0.11 (-0.24 – 0.03)	0.114	0.05 (-0.09 – 0.19)	0.482	-0.12 (-0.29 – 0.04)	0.146
Child's age						
Less than 3	Ref		Ref		Ref	
From 4 to 5	0.05 (-0.20 – 0.30)	0.703	0.30 (0.02 – 0.57)	0.035	0.34 (0.02 – 0.66)	0.036
From 6 to 11	0.28 (-0.09 – 0.65)	0.132	0.58 (0.18 – 0.98)	0.005	0.73 (0.27 – 1.20)	0.002
From 12 to 14	0.46 (-0.02 – 0.95)	0.062	0.70 (0.17 – 1.23)	0.01	0.63 (0.01 – 1.25)	0.047
From 15 to 18	0.37 (-0.20 – 0.94)	0.207	0.87 (0.25 – 1.50)	0.006	0.75 (0.02 – 1.48)	0.043
Child's education						
High school	Ref		Ref		Ref	
Elementary school	0.37 (-0.12 – 0.85)	0.139	0.60 (0.07 – 1.13)	0.027	0.50 (-0.12 – 1.12)	0.113
Middle school	0.14 (-0.25 – 0.52)	0.487	0.21 (-0.21 – 0.63)	0.324	0.17 (-0.33 – 0.66)	0.507
Kindergarten	0.53 (-0.03 – 1.10)	0.064	0.84 (0.23 – 1.46)	0.007	0.85 (0.13 – 1.57)	0.021
Preschool	0.59 (0.03 – 1.14)	0.038	0.98 (0.38 – 1.58)	0.001	0.92 (0.22 – 1.63)	0.01

## Discussion

Currently, there is an interest in assessing the impact of the COVID-19 pandemic on the mental health of adolescents and children. This interest can be explained by two main reasons. First, an increasing trend was observed in mental health problems among adolescents worldwide in general. There are also major gaps in the management of such problems. The observed increasing trend was even observed before the start of the COVID-19 pandemic. For example, the data from the National Survey on Drug Use and Health showed that 13.3% of adolescents aged 12 - 17 years old in the US suffered from one episode of major depressive disorder or more in 2017. Moreover, 60.1% of the affected individuals did not receive treatment for their illness [14]. Second, it has been shown that a link exists between viral diseases such as Influenza A and mental disorders such as anxiety and depression [15]. Thus, it can be expected that the current crisis would negatively impact the mental health of children and adolescents. The current study was developed to investigate the psychiatric impact of the COVID-19 pandemic and the associated quarantine on the mental health of children and adolescents in Saudi Arabia to identify gaps and possible solutions for such problems if such problems existed.

Income loss and economic issues can lead to stress and subsequent marital conflicts [16]. Moreover, quarantine can lead to lower privacy and freedom, which can cause a higher prevalence of stress [17]. Increased domestic violence can significantly affect the mental health of children and adolescents [18,19] and can create long-term consequences [20]. In the current study, we found a statistically significant positive correlation between child hitting and the increased prevalence of psychiatric problems which supports the previous findings.

Children and adolescents are at increased risk of prolonged and massive restriction in social connections due to quarantine. Moreover, families have to re-organize and adapt to new situations, such as quarantine and physical distancing. Temporary closure of schools has led to an increase in home-schooling, distance-learning, and challenges to food distribution [21].

Our results showed that 30% of the included children and adolescents were irritable to an extent that was higher than that before the quarantine and that 18% were more anxious after the quarantine than before it. These results are consistent with what was observed in a recent study which included children and adolescents from Spain and Italy. The study was conducted to assess the mental health of children and adolescents in Spain and Italy during the quarantine and used a data collection tool that was adopted in the current study. Results showed that 11.4% of the Spanish and Italian parents thought that family coexistence was not feasible during the quarantine. Nonetheless, the majority of the parents thought that COVID-19 was serious, and 33% of them reported being stressed or very stressed, although the difference between countries was not statistically significant [2].

Interestingly, the study reported an association between parents' perception of COVID-19 and their children's psychiatric symptoms during the quarantine. Their results showed that parents who reported high levels of stress were more likely to report more emotional problems in their children [9]. These findings are similar to what was reported in the current study where families whose income was affected by the pandemic reported higher change in the mental state of their children. Moreover, the study showed that higher levels of parental stress were related to more use of screens, less time of physical activity, and fewer hours of children's sleep. These results were also replicated in the current study as we showed that punishment threats and child hitting, usually markers of parents' stress, were associated with a higher degree of restlessness as well as anxiety, as well as a higher number of hours spent watching TV by the children. Our results are also consistent with the results from a Chinese study which showed that one-quarter of school-age Chinese children reported depressive symptoms. The results also showed that 19% of them reported anxiety symptoms 34 days after the quarantine was imposed as a preventive measure against COVID-19 [1].

Our results were also in line with another cross-sectional study that was conducted on 8079 Chinese students aged 12-18 years old during the duration of the pandemic. An online survey was used to collect the needed data. Depression and anxiety symptoms were assessed using the Patient Health Questionnaire (PHQ-9) and the Generalized Anxiety Disorder (GAD-7) questionnaire, respectively. Both of which were not used in the current study, the prevalence of depressive symptoms, anxiety symptoms, and their combination was 43.7%, 37.4%, and 31.3%, respectively. Multivariate analysis showed that females were at a higher risk of depression and anxiety than males [9]. Our results were not similar to those although an association was observed between gender and irritability/angriness domain, although the results were not similar for the other two domains. Nonetheless, these results can be explained by the fact that we did not use GAD-7 and PHQ-9 for data collection.

In terms of grades, senior high school was a risk factor for depressive and anxiety symptoms; the higher the grade, the greater the prevalence of depressive and anxiety symptoms. Our findings show there is a high prevalence of psychiatric health problems among adolescents, which are negatively associated with the level of awareness of COVID-19. These findings suggest that the government needs to pay more attention to psychiatric health among adolescents while combating COVID-19 [9].

On the positive side, only 9% of the parents in the current study thought that their children had behavioral problems during the quarantine to an extent greater than that observed before the quarantine. Our results also showed that children with family members who were infected with COVID-19 were more likely to show a change in mental status. Thus, parents are currently under pressure to keep jobs and businesses running, work from home, as well as support, educate and comfort their children, all at the same time. It can be expected that many families would not have the ability to restrict or isolate physically, with economic realities overcoming public health prevention efforts. In addition, parents also carry the role of explaining the pandemic and its effects on their children. They also have an important role in managing and containing the fear and anxiety that accompanies everyone in the family during these uncertain times.

The efforts to study the association between pandemics (previous pandemics and current COVID-19 pandemic) on the psychiatric problems in children and adolescents were summarized in a recent systematic review. Not only did the review assess the association between the Covid-19 pandemic on children's and adolescents' mental health, but it also evaluated the appropriateness of various interventions used during previous and the current COVID-19 pandemic in improving the mental health of children and adolescents. The review included 18 studies. Stress, social and risky behavioral problems (such as suicide, drug abuse, and academic issues) were reported in the majority of the included studies. Regarding interventions, support services, art-based programs, and clinician-led mental health and psychosocial services have been shown to positively target the mental health issues among children and adolescents [22].

The impact of these psychiatric problems can be devastating on children and adolescents as the currently adopted medical system prioritizes care for the most ill patients and those who are at high risk of death, while areas such as mental health have received less focused attention, especially in developing countries [23]. In addition, the Center for Disease Control (CDC) and Prevention reported an increase in the rates of US persistent sadness or hopelessness among high school students over the course of years (from 26% in 2009 to 37% in 2019). The rates of more serious problems such as suicide ideation and suicide planning increased from 14% to 19% and from 11% to 16%, respectively. Ultimately, the rate of suicide attempts increased from 6% to 9% [24]. Children and adolescents at risk should receive some form of special attention. Programs that provide children with psychosocial skills could help them cope with the COVID-19 situation [2]. Pediatric healthcare workers should receive training in order to detect children's psychiatric problems [25].

Managing such problems can be challenging during the quarantine as schools, aftercare programs, and some methods of high-risk treatment, such as day treatment, were temporarily closed. Paradoxically, this imposed quarantine led to the increase of interest in using strategies such as telehealth so that mental health professionals can continue to serve their patients in a creative way. The pandemic has also led to an increase in the use of telepsychiatry in dramatic ways that even early adopters of virtual strategies couldn't have predicted [26].

As expected, routines also changed during the quarantine for the children of both countries, spending more time using screens, spending less time doing physical activity, and sleeping more hours. Indeed, we reported that 45% of the children played video games to a greater extent after the quarantine and that 33% watched TV to a greater extent. In accordance with a review of studies that observed differences in habits between weekdays and vacation days, the present study found more healthy routines in youth before the quarantine [27].

In our study, we showed that one-third of the respondents suffered from boredom to a greater extent after the quarantine and that one-half played video games and watched TV to a greater extent after the quarantine. Brook and colleagues evaluated the psychological effect of the quarantine and ways to reduce the accompanying negative psychological effects such as confusion, post-traumatic stress, and anger. They showed that frustration and boredom may predict conflicts and stress within the family. The need for families to work, the unknown duration of the quarantine, the need to support children and provide distance-learning efforts may lead to long-lasting mental health problems. Parents also need to manage the effect of the pandemic and probable life loss sustained by families and communities [28].

Responses of children and adolescents to a crisis situation are defined by many important factors such as the physical and mental health issues, prior exposure to emergencies, cultural background of the respondents, and the socioeconomic circumstances of the family.

## Conclusions

COVID-19 has caused increased stress on families, especially children and adolescents who are vulnerable populations. Our findings show that the COVID-19 pandemic can affect the mental health of children and adolescents in Saudi Arabia. We showed that parental stress is a predictor of psychiatric problems, which, if unaddressed, can cause child maltreatment and greater psychological distress. Training parents and community healthcare providers to evaluate and address mental health problems in children and adolescents can act as the first line of support for parents and their children. As the battle continues against COVID-19, identifying and responding to mental health in children and adolescents is more important than before. By identifying mental health needs and possible interventions, the harmful medium-and long-term outcomes for individuals, families can be mitigated. Thus, it is important that resources are directed towards evidence-based practices to address the mental health issues that the COVID-19 pandemic has caused.
